# Transformation of Nano-Size Titanium Dioxide Particles in the Gastrointestinal Tract and Its Role in the Transfer of Nanoparticles through the Intestinal Barrier

**DOI:** 10.3390/ijms241914911

**Published:** 2023-10-05

**Authors:** M. S. Ryabtseva, S. F. Umanskaya, M. A. Shevchenko, V. S. Krivobok, A. V. Kolobov, A. A. Nastulyavichus, S. I. Chentsov, V. D. Sibirtsev

**Affiliations:** 1 P. N. Lebedev Physical Institute, Russian Academy of Sciences, Leninsky Prospect 53, Moscow 119991, Russia; umanskaya@lebedev.ru (S.F.U.); mishev87@lebedev.ru (M.A.S.); krivobokvs@lebedev.ru (V.S.K.); kolobov@lebedev.ru (A.V.K.); nastulyavichusaa@lebedev.ru (A.A.N.); chencovsi@lebedev.ru (S.I.C.); 2 Department of Veterinary Medicine, Institute of Veterinary, Veterinary-Sanitary Examination and Agricultural Safety, Russian Biotechnological University, Volokolamskoe Highway 11, Moscow 125080, Russia; sibircev_vd@mail.ru

**Keywords:** nanotechnology, nanoparticles, titanium dioxide, gastrointestinal tract, histohematic transport

## Abstract

In this work, the size transformation of the TiO_2_ nanofraction from pharmaceutical grade E171 powder was studied during its transit through the gastrointestinal tract (GIT). It was shown that pharmaceutical-grade TiO_2_ powder contained about 0.68% (*w*/*w*) of particles smaller than 240 nm in diameter. In the observed GIT transit process the TiO_2_ nanoparticles were agglomerated up to 150–200 nm in simulated salivary fluid, with gradual agglomerate enlargement up to 300–600 nm and more than 1 micron in simulated gastric fluid. In the intestinal fluid the reverse process occurred, involving a decrease of agglomerates accompanied by the formation of a small fraction with ~50 nm average size. This fraction can be further involved in the histohematic transport process. The acidity degree (pH) and mineral composition of solutions, as well as the transit speed along the gastrointestinal tract, influence the nature of the particle transformation significantly. The rapid passing between the gastrointestinal tract sections creates conditions for a decrease in part of the TiO_2_ particles, up to 100 nm, and may be associated with the violation of the structural and functional integrity of the intestinal mucus layer.

## 1. Introduction

Nanotechnology achievements have become an integral part of human life. Today, there is no doubt about the systemic availability of many types of nanoparticles (NPs), including TiO_2_, SiO_2_, Ag, Au, grapheme and others, for alimentary consumption [1,2,3,4,5], which opens up a significant potential for the development of noninvasive nanoteranostics methods. Nevertheless, the fact of the alimentary availability of nanostructures requires close attention due to their safety issues.

Titanium dioxide has great popularity as a white dye and is used in the pharmaceutical industry. It demonstrates very low solubility in aqueous solution, is excreted from the body in transit and does not cause undesirable effects, as it was thought until recently. With the development of the nanotechnology cluster, a significant number of studies on the safety of titanium dioxide have been carried out, revealing its toxic potential. In most studies, where the bioavailability and toxic effect have been studied, they are associated with the action of the nano-sized fraction of TiO_2_ [1,5,6,7,8,9]. Such TiO_2_ NPs can be found in the vast majority of food and pharmaceutical grade powders [10]. A lot of works are devoted to the study of the cytotoxic effects of TiO_2_ NPs in vitro. In the works, where the NPs’ toxic effect has been convincingly demonstrated, the oxidative stress is considered the main mechanism of the cellular and molecular toxicity of NPs [11,12].

In contrast to in vitro studies, the results of in vivo toxicity are inconsistent [1,9,13,14,15,16,17,18,19,20,21]. Analysis of the reasons for the inconsistent results of most in vivo studies is limited to general ideas about the NPs’ size, concentration and exposure time [22]. However, there may be other exogenous and endogenous factors, with no obvious influence. For example, recent studies [23] have shown a significant transformation of the NPs’ surface in biological fluids due to the adsorption of molecules on their surface and the role of this transformation in the manifestation of toxic properties [24].

The gastrointestinal tract (GIT) environment is a multi-component colloidal system of mineral and organic compounds [25]. This system is characterized by variability between the GIT parts and over time. Such an environment has a significant potential for the transformation of NPs, and thereby ensures the operation of the first link of the intestinal barrier. At this stage, a rough size differentiation occurs, according to the particles’ ability to penetrate into the underlying layers, as in a system of sieves. The nanofraction aggregates’ size governs their penetration depth into the intestinal barrier and the possibility of transport into the body’s internal environment [26]. The modern scientific literature indicates that the mechanisms of penetration through the GIT into the blood are radically different for different sizes of particles. In which, for different penetration mechanisms different toxicity scenarios should be expected [26].

Insufficient understanding of the molecular processes underlying the NPs’ biotransformation that occurs during their transit through the GIT leads to inconsistency in the toxicological studies data. Additionally, in the absence of pronounced acute toxic effects, it does not allow us to unambiguously trace the relationship between the effects of NPs and specific biochemical and physiological changes occurring in the body. The studying of processes that occur during TiO_2_ particles’ transit through the GIT makes research in this field relevant for understanding the role of these processes in ensuring bioavailability as well as their biological action.

In the present study, we sought to gain insight into the processes occurring to the TiO_2_ NPs when crossing the gastrointestinal tract in order to trace their relationship with previously identified biological effects. We used TiO_2_ NPs, extracted from pharmaceutical grade powder, which are consumed daily in foods and drugs. Due to a number of methodological limitations, this area remains underrepresented in biomedical research [27]. All of this determines the novelty of this study. The study of these processes will be useful for obtaining fundamental information about the mechanisms of TiO_2_ NPs’ transport through the intestinal barrier, and for understanding their toxicity pathogenesis.

## 2. Results

### 2.1. NPs Characterization

**Figure 1:**
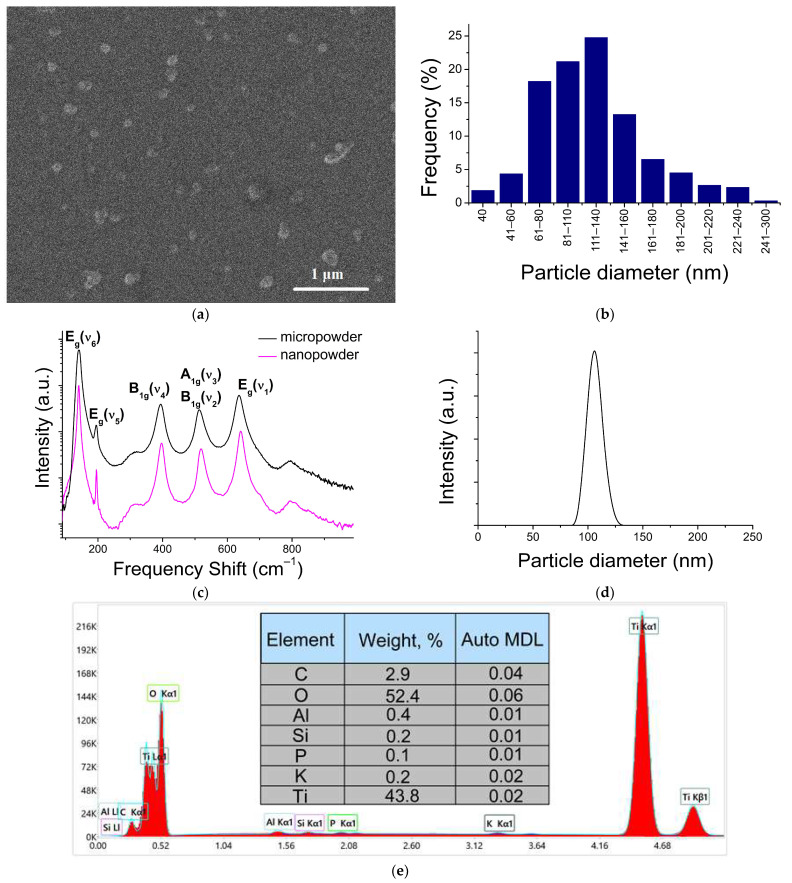
Characteristics of the TiO_2_ nanofraction: (**a**) TiO_2_ nanofraction SEM image; (**b**) TiO_2_ NPs’ size distribution obtained using SEM data; (**c**) Raman spectra of nano- and microfraction of the E171 powder; (**d**) NPs’ size distribution obtained using DLS measurements; (**e**) typical EDX spectrum of the TiO_2_ nanofraction of EDX data.

SEM images showed that the E171 nanofraction mainly consists of separate spherical TiO_2_ NPs of ~40–240 nm in diameter (Figure 1a,b). Among the single particles, large agglomerates (1–2 per 150 in the field of view) were visually detected. These particles were about 300 nm in size, and consisted of several single particles 130–150 nm in size.

The NPs’ size distribution was also studied via dynamic light scattering (DLS) (Figure 1d), and is qualitatively consistent with the SEM data. It should be noted that some of the difference in the particles’ distributions presented in Figure 1b,d is associated with the methods’ specificity. The DLS method is not able to resolve relatively small particles against a background of large ones, due to a sharp increase in the scattering cross-section with increasing particle size (~r^6^). This is also noted in [27]. On the contrary, the SEM method leads to the appearance of a “tail” from the side of large particles. This is due to the aggregation of particles during the drying of the colloidal solution on the substrate.

The Raman spectra of the E171 powder and the nanoparticles separated from its nanofraction were identical and corresponded to the allowed first-order scattering processes involving long-wavelength optical phonons of the TiO_2_ anatase phase (Figure 1c).

An EDX analysis was performed to confirm the composition. Both fractions (macro-disperse powder and nanofraction) consisted mainly of titanium and oxygen, in addition to which peaks corresponding to Al, Si, P, K, and C were stably present in the spectrum (Figure 1e). Presumably, Al and Si are contained in the composition of the studied powder in the form of the corresponding oxides. Those oxides can also play the role of an anti-caking agent. The proportion of TiO_2_ NPs with a diameter less than 450 nm (detected via the ICP-MS method) was about 0.68% by weight of the total weight in the E171 additive powder.

### 2.2. NPs Transformation at Separate Stages of Digestion

**Figure 2:**
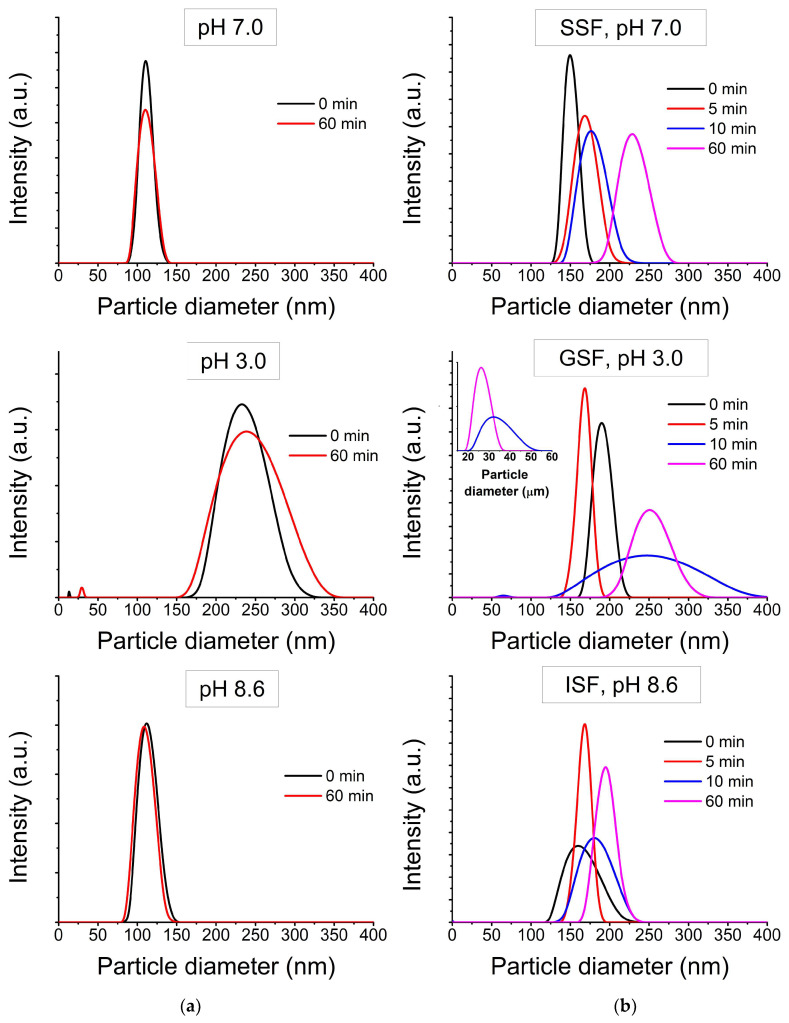
Transformation of TiO_2_ NPs’ size (**a**) in deionized water at various pH values; (**b**) in GIT simulation liquids.

Figure 2 shows the distribution of the hydrodynamic diameters of NPs in deionized water with a pH corresponding to the GIT stages (Figure 2a) and in simulated GIT fluids (Figure 2b). In both cases, the particles behaved in a similar way, but, in highly mineralized solutions, the average particle size was larger. In deionized water with pH 7, the registered particle diameter was about 120 nm. In deionized water with pH 8.6, the registered particle diameter was about 110–120 nm. In both cases, the particle sizes remained stable over time. In deionized water with pH 3.0, the particle size was registered in two ranges: about 15 nm and about 200–300 nm, and became larger with time—about 30 nm and about 225–350 nm, accordingly, after an hour. In SSF, at pH 7, the registered particle diameter was about 150–200 nm. In ISF, at pH 8.6, the average registered particle size was about 170 nm. In both simulation solutions, the sizes of particle agglomerates remained stable over time. In GSF, at pH 3.0, the particle size was not stable over time. The first time, the particle size was recorded in two ranges: about 70 nm and about 170–250 nm, and they grew larger with time. The obtained results are consistent with the work [28], where the dependence of the particles’ colloidal stability on the solution’s pH was shown, and are partially consistent with the work [27]. In Ref. [27] small changes in the size distribution of TiO_2_ NPs were observed in saliva, while in simulated gastric fluid there was a sharp increase in hydrodynamic size.

### 2.3. NPs’ Transformation in Conditions of Sequential Passing through a GIT Section

**Figure 3:**
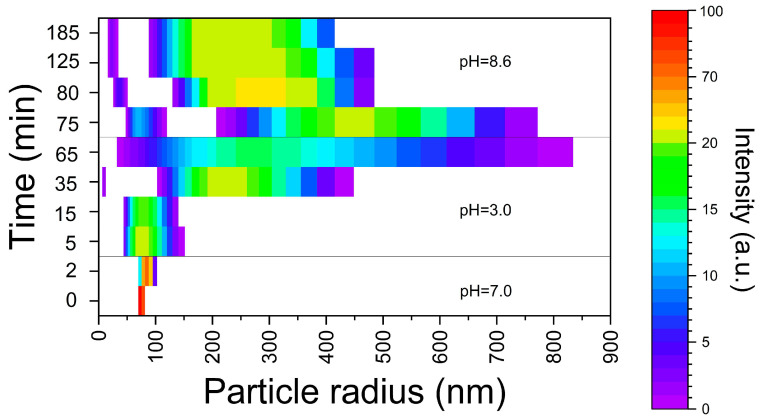
Transformation of TiO_2_ NPs’ size in conditions of sequential passing through GIT section (using DLS data).

The dynamics of the E171 additive nano-sized fraction’s transformation in conditions of sequential passing through a GIT sections are shown in Figure 3. The NPs solution in deionized water contained particles with an average recorded diameter of 115 nm. During 2 min incubation with SSF at pH 7.0, the particles’ size enlargement was up to 150–200 nm. After transferring to a stomach acidic environment, there was a further enlargement of the particles. At the same time, after 20–30 min in the simulated environment of the stomach, in addition to the particles larger than 300 nm, particles with diameter about 20 nm were registered. By the end of an hour the agglomerates’ size reached more than 1 μm. The small fraction was not detected. With the further transfer of particle agglomerates into the ISF, with a pH of 8.6, the destruction of the agglomerates gradually occurred. After 60 min, particles were detected in two size ranges: ~50 (33–67) nm and 300–600 nm. By the end of 2 h of incubation, the particle size distribution remained at the same level.

### 2.4. Gastrointestinal Transit Rate

Eight series of rat gastrointestinal tract X-ray images with contrast agent were selected for analysis. All cases met the requirements. The age of the patients ranged from 3 to 12 months. In all cases, barium sulfate (Bar-VIPS, VIPS-MED, Fryazino, Russia) was used as an X-ray contrast agent. The solvent was distilled water. It was introduced in an intragastric (i.g.) manner, atraumatically, through a gastric tube, in a volume of 10.0 mL/kg body weight.

**Figure 4:**
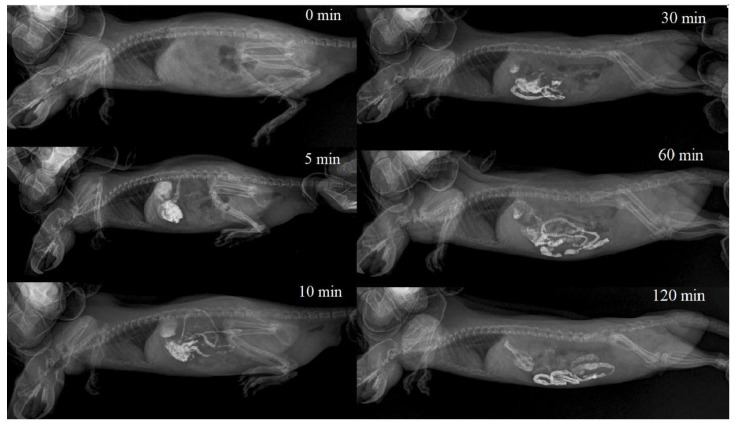
Barium sulfate colloidal solution gastrointestinal transit rate in a rat. A series of consecutive X-ray images.

Powder Bar-VIPS (VIPS-MED, Fryazino, Russia) contains particles of barium sulfate in the 0.2–3.5 μm size range. When prepared according to the instructions for medical use, the powder forms a stable suspension with a 1.0–8.0 µm particle size (the percentage of the particles with a size of 1.0–2.0 µm is about 60%) [29]. In all cases, immediately after the i.g. administration, the colloidal solution of barium sulfate completely filled the stomach volume, and partially passed into the duodenum. After 30 min, barium sulfate was visualized mainly in the glandular (pyloric) part of the stomach, duodenum and jejunum. In the non-glandular part of the stomach, it was practically not visualized. By the end of the first hour, barium sulfate was visualized in the stomach in trace amounts, which remained the same by the end of the second hour after administration. During this period, the bulk of the X-ray contrast agent was distributed in the intestines, reaching the ascending loop of the ileum, before the cecum. A typical X-ray image series is shown in Figure 4.

## 3. Discussion

In this research, it was shown that the TiO_2_ nano-sized fraction of E171 undergoes dynamic reversible transformation under GIT conditions. The environment composition has a significant effect on the dimensional characteristics of the nanofraction aggregates formed under the conditions of the GIT. As it follows from our data, the nature as well as the rate of the TiO_2_ NPs’ transformation processes involved are governed by the chyme consistency, its compounds and the duration of their stay in the GIT. The mineral composition of the GIT environment leads to the formation of larger particles compared to a low-mineralized solution with the same pH. Under low-mineralized conditions with variable pH, the concentration of free H^+^ ions has the greatest effect on aggregative stability. The high concentration of H^+^ ions leads to a decrease in the ζ potential due to the contraction of the electrical double layer. Electrostatic repulsion is weakened and particles stick together. An increase in the ionic strength of the solution also compresses the electrical double layer on the surface of TiO_2_ NPs in accordance with the Deryagin–Landau–Verwey–Overbeck theory. In this case, divalent ions can more effectively compress the double electric layer than monovalent ions, due to the Schulze–Hardy rule. In terms of charge shielding, compared to monovalent ions, divalent ions have a higher charge density and can induce nano-TiO_2_ aggregation accompanied by sedimentation at a lower ionic strength. It is important that under the conditions of each GIT section particles larger than 100 nm are formed, whereas passing between GIT sections creates the conditions for the formation of particles < 100 nm. As it follows from Figure 3, in our model in vitro experiments this fraction was unambiguously observed in the first 20–30 min after passing from the SSF to GSF (the particles’ size was about 20 nm), and later after passing from the GSF to ISF. In this case particle agglomerates were destroyed, and particles with ~50 nm size were formed among others (Figure 3).

In the intestine the main absorption of food substrates is observed. Due to the interaction with chyme and elements of intestinal mucus, the size differentiation of insoluble particles occurs according to their ability to penetrate into the deep layers of the intestinal barrier. According to [30], which describes the size characteristics of pores in the structural assembly of the intestinal mucus, colloidal particles smaller than 100 nm diffuse efficiently through the mucus layer, while larger particles (>500 nm) demonstrate limited diffusion. Those particles that have a chance to penetrate deeper layers of the mucus due to their size can interact with its components [31,32]. At least five main pathways for intracellular transport of NPs have been shown in vitro [26]: phagocytosis (for large particles, initiated by opsonization), clathrin-mediated endocytosis (for particles < 100–150 nm), caveolin-mediated endocytosis (for particles < 50–80 nm), clathrin/caveolae-independent endocytosis and macropinocytosis (pinocytosis vesicle about 0.2–5 μm). Taking into account size restrictions, TiO_2_ NPs with a particle size less than 100 nm can be further involved in the histohematic transport process so they have high bioavailability potential. The number of these particles is about ~1.3% of all particles of the nanometer range and, accordingly, about 10^−5^% of the total weight of food additives.

Unlike the single-chamber in vitro digestion model, where changes occur simultaneously and spread throughout the entire reaction volume, processes occurring in the GIT (in vivo) are characterized by significantly greater variability, due to the portioned transfer of food between GIT parts, the gradual release of enzymes, mixing chyme due to peristaltic movements and absorption. This creates conditions for greater variability in the results of theTiO_2_ NPs’ transformation during their transit through the GIT. In this research, it was shown that in vitro particles’ aggregation in a simulated GIT environment was time dependent in few cases. To establish the actual time the particles spent in the rat GIT at standard protocol for in vivo acute toxicity, an assessment of the solution’s intestinal transport rate was carried out. As it follows from Figure 4, the colloidal solution starts to pass into the intestinal lumen immediately after i.g. administration, and the half-life of the liquid contents from the rat stomach is about 30 min, which is consistent with the data [33,34,35]. Thus, in the in vivo study, up to half of the injected volume of the TiO_2_ solution can be transferred to the intestine, bypassing interaction with gastric juice. A certain part of the solution volume reacts with gastric juice, but the interaction time is much less than 60 min. Taking into account these considerations, it should be expected that the portion of bioavailable nanoparticles may be less in the case of rapid passage through the gastrointestinal tract.

At the same time, in our previous research [5], significantly higher TiO_2_ accumulation in rats was shown (the dose and course duration were taken into account). This suggests that larger particles can also cross the intestinal barrier under conditions of long-term intake. A short period of interaction with gastric fluid and, probably, an uncompleted mixing process, may leave some “reaction sites” on the surface of the NPs free. Such particles, probably, more effectively realize their reactive potential in the intestinal lumen, where they bind ions (including Ca^2+^ and Mg^2+^) and intestinal mucus components. The mucin disruption may be associated with an increase in the permeability of the intestinal barrier and makes the underlying epithelial cells more vulnerable [36]. This is also partially consistent with toxicological data previously obtained [6]. Despite the absence of pronounced morphological changes in the intestinal mucosa, changes in a number of biochemical blood parameters may indirectly indicate a violation of the integrity of the mucin layer which requires additional research.

## 4. Materials and Methods

### 4.1. Materials

In this work, anatase TiO_2_ NPs, isolated from a E171 sample (Pretiox AV-01-FG, Přerov, Czech Republic) of food and pharmaceutical grade, were studied. All chemicals were at least an analytical grade, provided by Sigma Aldrich (St. Louis, MO, USA), unless otherwise noted. Deionized water was obtained using the EasyPure system (Thermo Scientific, Waltham, MA, USA).

### 4.2. NPs Extraction

For TiO_2_ NPs’ extraction from E171 powder, the dispersion of E171 food powder was prepared in deionized water at a concentration of 2 mg/mL. The samples were subjected to ultrasound in the ultrasonic bath “Sapphire” USV 1.3 (LLC Sapphire, Saint-Petersburg, Russia) at 35 kHz for 10 min at +23 °C, to reduce the role of particle aggregation. The micro-sized fraction was separated via centrifugation at 4500 rpm for 10 min. The supernatant was filtered through a filter with 450 nm pore size.

### 4.3. NPs’ Characterization

The crystallinity of particles was confirmed via Raman spectroscopy (RS) (Rammix 532R (EnSpectr, Moscow, Russia) coupled with Olympus CX-41 microscope (Olympus, Tokyo, Japan)). A CW laser operating at a wavelength of 532 nm was used as an excitation source. The spectral resolution was 4 cm^−1^.

Morphology and particle size were measured using a Tescan VEGA scanning electron microscope (SEM, TESCAN Essence, Brno, Czech Republic) with low–high vacuum functions, and an EDX chemical microanalysis module (Oxford Instruments, Oxford, UK). The images were taken at 5 keV accelerating voltage. A colloidal solution of the isolated nanofraction in deionized water was applied to a carbon substrate and dried in air for 12 h before analysis. Samples were tested without additional coating. The loading of particles in the analyzed area was no more than 10%. The chemical composition of the particles was determined via energy dispersive X-ray spectroscopy (EDX).

The particle size distribution was carried out by counting on electron micrographs. Counting was performed in 10 fields of view for three independent samples. The results were ranked in size ranges from 0 to 300 nm. The number of particles for each range was calculated in %. The total number of particles in the samples was taken as 100%.

The dynamic light scattering method (DLS) was used for confirming primary particles’ size (in deionized water) and for measuring NPs’ transformation in the GIT. Measurements were carried out via Photocor Compact-Z (Photocor, Moscow, Russia), at 23 °C. Three independent measurements were performed for each solution.

Quantitative determination of NPs’ mass fraction in the sample (NPs extract solution) was carried out via high-resolution mass spectrometry with ionization in inductively coupled plasma (ICP-MS) (Finnigan Element-2, Thermo Scientific, Waltham, MA, USA). Separation of the ions was carried out via an analyzer with double focusing—magnetic and electrostatic modes. The ions were detected via an electron multiplier, which remained linear in the range from 1 to 1 × 10^10^ ions per second. The measurement was carried out in an argon flow. Titanium was determined via the ^47^Ti isotope in the medium resolution mode to eliminate interference. For calibration, reference 68-element solutions for ICP-MS (ICP-MS-68A, solutions A and B) with element concentrations of 0.03–10 ppb were used. The detection limit for Ti was 0.0002 ppm. The error in 9 parallel measurements of each sample did not exceed 0.2%. Samples were decomposed by treating colloidal solutions with HF until the particles were completely dissolved. The measured samples were then diluted with 3% HNO_3_. The concentration of 10 ppb was used as an internal standard.

### 4.4. NPs’ Transformation in the GIT

To simulate the GIT environment in vitro, a static three-chamber model with constant ratios of components at each stage of digestion (oral, gastric, and small intestine phases of digestion) was used [25]. Digestive enzymes and Ca^2+^ ions were excluded from the simulation fluids. To obtain the target concentrations of salts in imitation liquids, the matrix solution of imitation liquids was prepared, taking into account the subsequent twofold dilution.

In the first series of experiments, the transformation of NPs at each stage of digestion was studied. The NPs solution was prepared as described before. For oral cavity simulation, a matrix solution of saliva-simulated fluid (SSF) was mixed with the NPs solution, in a ratio of 1:1. The pH of the solution was adjusted to 7.0 by adding the required amount of 1 M HCl. Interaction time was 2 min. For gastric simulation, the matrix solution of gastric-simulated fluid (GSF) was mixed with the NPs solution in a ratio of 1:1. The pH was adjusted to 3.0 using 1 M HCl. Interaction time was 60 min. For intestinal simulation a matrix solution of intestinal-simulated fluid (ISF) was mixed with the NPs solution in a ratio of 1:1. The pH was adjusted to 8.6 using 1 M NaOH. Interaction time was 60 min.

The measurements were carried out in comparison with NPs solutions in deionized water with similar pH values (3.0, 7.0, 8.6). The pH was adjusted with 1 M NaOH or 1 M HCl.

In the second series of experiments, the dynamics of the NPs’ transformation in conditions of sequential passing through each section of the GIT were simulated. For this, 5.0 mL of NPs solution was mixed with 5.0 mL of a SSF matrix solution; pH was 7.0, interaction time was 2 min. After that, 5.0 mL of the GSF matrix solution was added to 10 mL of the resulting solution. The pH was adjusted by adding the required amount of 1 M HCl to a physiological value of 3.0. The total volume of the solution was adjusted to 20.0 mL with deionized water. Interaction time was 60 min. After that, 10.0 mL of ISF matrix was added to 20.0 mL of the resulting gastric solution. The pH was adjusted to 8.6 using 1 M NaOH. The total volume of the solution was adjusted to 40.0 mL with deionized water. Interaction time was 120 min.

### 4.5. Gastrointestinal Transit Rate

To investigate the role of the food passing rate between GIT sections, we evaluated the time of gastric emptying and rat GIT transit rate. The assessment was carried out by comparing serial radiographic images performed after intragastric administration of an X-ray contrast colloidal solution with comparable particle size. The retrospective data from clinical X-ray studies of the rodents’ GIT were used. Rats’ GIT X-ray results, performed in 2022–2023 in Moscow veterinary clinics, with barium sulfate (Bar-VIPS, VIPS-MED, Russia) as an X-ray contrast agent, were selected for the analysis. Additional criteria for the selection were a volume of intragastric administration of 10 mL/kg; the ability to determine the time of a series of images; and the absence of gastrointestinal motility disorders in the conclusion of the X-ray study.

## 5. Conclusions

The investigation the TiO_2_ NPs’ transformation dynamics under conditions simulating the GIT have a fundamental role in understanding the mechanisms of the TiO_2_ NPs’ transport through the intestinal barrier and their toxicity pathogenesis. The obtained data indicate that the nanofraction of the food additive E171 undergoes a dynamic reversible transformation in the GIT environment. This process depends on many factors, including the active acidity, mineralization system, as well as the rate of passing through the GIT. In the process of transfer along the GIT, the nanofraction is not completely “neutralized”; a small portion of the particles is available for histohematic transport. Conditions for the formation of particles with high bioavailability potential (particle size < 100 nm) are created twice during the transition between sections of the GIT. The efficiency of the TiO_2_ NPs’ aggregation process apparently depends on the gastrointestinal transit rate. Faster transit between GIT sections creates conditions for a decrease in the portion of TiO_2_ particles up to 100 nm, and may be associated with a structural and functional integrity violation of the intestinal mucus layer. Thus, slightly different toxicity study protocols could be the reason for inconsistency in the data of the toxicological studies presented in the literature.

The increasing research interest to transformation of NPs under GIT conditions and the mechanisms of NPs’ transport through the intestinal barrier can be merged with toxicology areas of investigation. Such approaches can be exploited further to improve the safety of insoluble pharmaceutical- and food-grade powders with a nano-size fraction, and for developing new noninvasive nanoteranostics methods.

## Data Availability

Not applicable.
